# *Tunga penetrans* causing a rapidly progressing foot ulcer in a patient with uncontrolled type 2 diabetes mellitus

**DOI:** 10.1093/omcr/omac016

**Published:** 2022-03-16

**Authors:** Ashabilan A Ebrahim, Elisia P Mpango, Joseph A Temba, Zulfiqarali G Abbas, Fredirick L Mashili

**Affiliations:** Department of Physiology, Muhimbili University of Health and Allied Sciences (MUHAS), Dar es Salaam, Tanzania; Department of Surgery, Amana Regional Hospital, Dar es Salaam, Tanzania; Department of Physiology, Muhimbili University of Health and Allied Sciences (MUHAS), Dar es Salaam, Tanzania; Department of Medicine, Abbas Medical Centre (AMC), Dar es Salaam, Tanzania; Department of Physiology, Muhimbili University of Health and Allied Sciences (MUHAS), Dar es Salaam, Tanzania

## Abstract

Tungiasis is a parasitic disease resulting from infestation by a female flea *Tunga penetrans*. The parasites are endemic in the tropics and can infect patients with diabetes mellitus (DM). Augmented by uncontrolled hyperglycemia and pre-existing neuropathy, the parasite may trigger a locally spreading inflammation, which may aggravate the trauma introduced during its extraction, leading into a rapidly progressing foot ulcer. To the best of our knowledge, no such cases in patients with type 2 diabetes have ever been published from Tanzania and likely none worldwide. This case report shows that, in diabetic patients, the wound resulting from the extraction of *T. penetrans* may get infected and aggravated by the ongoing inflammatory reaction, rapidly evolve into limb-threatening condition and mortality. Preventive measures are necessary and should be emphasized in patients with DM. Studies are needed to increase our understanding of the pathophysiology, proper management and sequalae of ulcers of this nature.

## INTRODUCTION

Tungiasis refers to a cutaneous ectoparasitic infection of the skin with the female flea, *Tunga penetrans*. The disease is a fairly common infection in sub-Saharan Africa [1]. Infection often occurs in the lower limbs, and the infected site presents with severe inflammation and ulceration with secondary bacterial infections that may lead to sepsis, osteomyelitis and eventually gangrene [2].

Diabetic patients are at risk of developing foot ulcers from a combination of hyperglycemia, trauma, underlying diabetic peripheral neuropathy (DPN) and peripheral arterial disease (PAD) [3]. In diabetes, *T. penetrans* infection and accompanied trauma following its extraction may predispose to rapidly progressing ulcers [3]. We report such a case in a 50-year-old diabetic woman of African descent who presented with an ulcer on the plantar surface of the first metatarsal-phalangeal joint.

## CASE REPORT

A 50-year-old woman presented to us with a 2-week history of a progressing ulcer on her right foot following a cutaneous infestation by a sandflea, *T. penetrans.* The patient had traveled to a known tungiasis endemic area in Tanzania [4]. She was a small-scale businesswoman, known type 2 diabetic for 5 years and not on regular medication.

She reported to have noticed a whitish ‘nodular’ lesion at the tip of the second toe, with no accompanying pain or itching. Removal of the flea using nonsterile sharps was done, leaving an initial small wound. She later observed a similar lesion across the first right metatarsal-phalangeal joint, which was also punctured to remove another flea. Following the removal, an abscess developed around the wounds, extended to involve the medial and lateral aspects of the second and big toes, respectively. At this point, there was accompanying pain and swelling of the foot. The abscess ruptured spontaneously turning into an ulcer that occupied the medial and lateral aspects of the second and big toes, respectively.

Within 2 weeks, the wound had progressed into a foul-smelling ulcer. It extended to the plantar aspect of the big toe across the metatarsal phalangeal joint and eventually involved the antero-medial aspect of the right foot. In parallel, she experienced fever, generalized malaise and episodes of fatigue.

**Figure 1:**
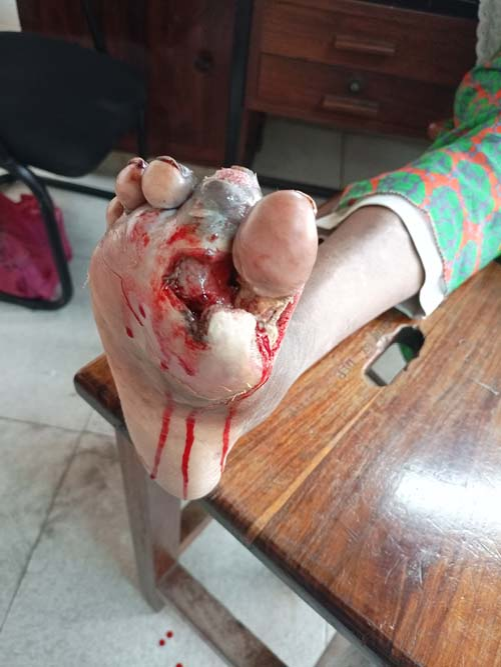
Deep-seated ulcer on the plantar surface between the first and second metatarsal-phalangeal joints (5 × 3 cm) extending to the medial side of the right foot.

**Figure 2:**
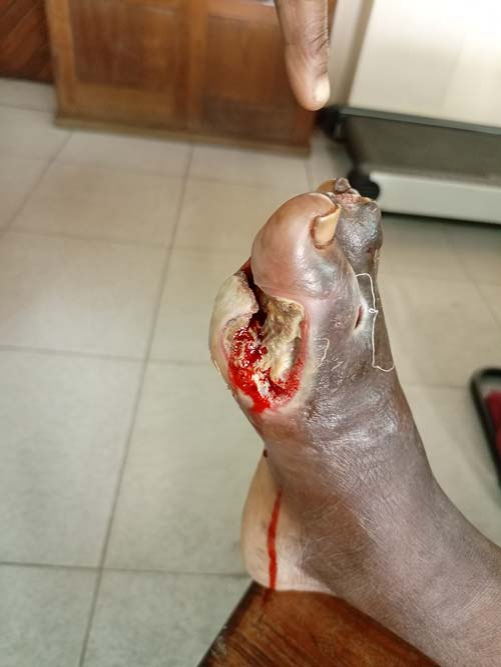
A narrow extension across the first metatarsal phalangeal joint with an expansion on the medial side (4 × 3 cm).

On examination, there was a deep-seated foul-smelling ulcer on the plantar surface between the first and second metatarsal-phalangeal joint extending between the second and big toes (5 × 2cm) (Fig. 1). Also, there was a narrow extension across the first metatarsal phalangeal joint with an expansion on the medial side (4 × 3 cm) (Fig. 2).

Examination of the peripheral arterial function using an automated device (Smartdop XT, Kodymedics, India) revealed bilateral impairments in peripheral arterial function, and these were letter confirmed by Doppler studies. The patient had moderate DPN as measured by vibration perception threshold (25v). Her random blood glucose and glycated hemoglobin (HbA1C) were 18 mmol/dl and 8.9%, respectively. She was diagnosed to have a neuro-ischemic diabetic foot ulcer secondary to *T. penetrans* and was admitted for further management.

Patient’s blood glucose was controlled using insulin, and infection was treated empirically with initial parenteral (iv Ceftriaxone and Metronidazole), then oral antibiotics (Amoxicillin and Metronidazole) and dressing. Symptoms and signs of infection subsided 3 days later, and her blood glucose was between 6 and 8 mmol/l in three consecutive days. Nevertheless, despite successive infection and blood glucose control, the ulcer progressively increased in size. Due to worsening peripheral arterial status (infected toes turning gangrenous), the patient was scheduled for disarticulation of the two affected toes.

Following the decision, counseling was done. However, after repeated attempts, she firmly refused to have the procedure and voluntarily requested to be discharged from the hospital. She continued with regular dressing and oral antibiotics (amoxicillin) while at home. One week later, the patient was brought back and readmitted at the Emergency Department. She presented with fever and loss of consciousness and, having been stabilized, she was rushed to a tertiary hospital for intensive care. The patient was managed at the intensive care and apparently died 24 hours later.

## DISCUSSION

Tungiasis is a parasitic disease common and endemic in specific localities in the tropics [1, 2]. Being common in the tropics, it may coexist with diabetes mellitus (DM). Despite logical speculations from the pathophysiology of the two diseases pointing to their synergy in causing diabetic foot ulcers [1, 5–7], evidence for this concurrence is still lacking. To the best of our knowledge, we report for the first time, a case of rapidly progressing foot ulcer following infestation by *T. penetrans* in a patient with poorly controlled DM.

This case, with poor glucose control due to neglected diabetes treatment, presented with severe DPN and PAD. Together, these co-morbidities might have synergistically played a role and facilitated the rapid evolvement and advancement of the ulcer [1, 6, 7]. The wound was caused by trauma that resulted from the extraction of the parasite, while its progression was aggravated by the widespread inflammation and infection possibly caused by the parasite (Table 1).

**Table 1:** Full blood count results showing high total white blood cells (WBCs) signifying infection. Neutrophil predominance points toward bacterial infection. Additionally, there is microcytic hypochromic anemia

Indices	Absolute count	Reference range	%	Interpretation
Differential count				
WBC	16.3 × 10^3/μl	4.5–11		H
NEU	13.6	2–9	83.5%	H
LYM	1.11	1–3.3	6.82%	N
MONO	1.43	0–1	8.79%	H
EOS	0.031	0–0.7	0.192%	H
BASO	0.122	0–0.15	0.746%	N
Hematology				
RBC	3.61 × 10^6/μl	3.8–5.2 × 10^6/μl		N
HGB	9.76 g/dl	11–17 g/dl		L
HCT	27.8%	36–48%		L
MCV	77.0 fl	81–102 fl		L
MCH	27.1 pg	28–35 pg		L
MCHC	35.1 g/dl	31–36 g/dl		N
RDW	13.4%	11.6–14.8%		N
PLT	439 × 10^3/μl	140–450 × 10^3/μl		N
MPV	4.05 fl	7–12 fl		L

The reported patient noticed an already enlarged nodule with a grown flea, with no previous itching and pain. In tungiasis, growth of the flea usually triggers acute inflammatory reaction and cause itching and pain [2]. In this patient, however, the pre-existing DPN likely obscured these symptoms and was the reason for the delay in noticing the existence of the parasite. Evidence from previous reports show that *T. penetrans* infestation can introduce both aerobic and anaerobic bacteria [8]. Additionally, the use of non-sterile sharps to extract the parasite may also introduce bacteria [8]. Therefore, it is likely that the flea, unsterile sharps, or both, introduced bacteria into the wound and caused infection.

Moreover, this patient had PAD that caused ischemia and was advancing to cause gangrene. PAD is fairly common in DM [9]. Nevertheless, *T. penetrans* is implicated in causing ischemia and gangrene [2] and could have contributed to its progression. Due to advancing ischemia, evident by early signs of gangrene, the two affected toes were scheduled for disarticulation.

This case shows that tungiasis can trigger a rapid development and progression of a wound in a patient with uncontrolled DM. Effective foot care should be emphasized in those with DM and residing in tungiasis-endemic areas. Furthermore, in the tropics, research to uncover the effective and appropriate management of tungiasis-related foot ulcers in patient with DM is mandated.
